# Culture and Borderline Personality Disorder in India

**DOI:** 10.3389/fpsyg.2020.00714

**Published:** 2020-04-21

**Authors:** Shalini Choudhary, Rashmi Gupta

**Affiliations:** ^1^Shaheed Rajguru College of Applied Sciences for Women, University of Delhi, New Delhi, India; ^2^Cognitive and Behavioral Neuroscience Laboratory, Department of Humanities and Social Sciences, Indian Institute of Technology, Mumbai, India

**Keywords:** personality functioning within cultural context, normal and abnormal personality, borderline personality disorder, culture and BPD, BPD in India

## Abstract

The present paper discusses how cultural context can be used as an essential tool in the diagnosis of mental disorders as well as in segregating individuals with normal or abnormal personality functioning. Further, such information about the culture can be used to diagnose individuals specifically with borderline personality disorder (BPD). BPD is a mental illness marked by impulsivity and instability in interpersonal relationships, moods, and self-image. We discuss how culture shapes patterns of behavior in the individual with BPD. An extensive review of manifestation of BPD in eastern countries suggests that culture and social norms are the two crucial factors, which can differentiate normal and abnormal behavioral patterns in BPD. For example, the social norms shape and give directions to our interpersonal functioning, emotions are experienced within the cultural context and expressed accordingly, and self is a reflection and representation of one’s culture. Hence, these constructs of BPD (such as interpersonal functioning, emotions, and self) indicate that they are experienced differently in different cultures and vary on the continuum of normal to abnormal personality functioning. The cultural manifestation of BPD helps to understand the specific profile of the three constructs of BPD in an eastern culture like India. A synthesis of studies on prevalence, development, and symptom presentation of BPD in the Indian context is emphasized to support the personality functioning within the constructs of BPD. The literature review indicates that BPD research in India is based only on a few cases and a tiny sample of such patients. However, studies on BPD in the cultural context are minimal and incomprehensive. Hence, it suggests the importance of more empirical studies concerning the appropriate diagnosis of BPD within the cultural context of India.

## Introduction

Culture is understood in terms of a context. Context refers to the social and environmental specificities of the local world in which individuals and groups develop. These specificities or specific elements include (1) subjective perceptions, which indicate systems for interpreting human interactions and experience; (2) social structures that limit and allow diverse possibilities of activity and access to resources across individuals in a particular society; (3) the local physical environment, which sets standards for the group’s interactions with natural resources and technology; as well as (4) the individual situations, which are variable across persons and over time (Kirmayer and Ryder, 2016). Hence, cultural context provides a framework for individuals to form certain patterns of thinking, feeling, and behaving in their local worlds. These patterns can be in accordance with the context indicating normal patterns, or they can be discordant or adverse to such context indicating abnormal patterns of thinking, feeling, and behaving. According to Eshun and Gurung (2009), culture contains beliefs, principles, standards, activities, and symbols which, are environmentally acquired societal experiences. Moleiro (2018) has further stated that these cultural contexts shape the threshold of distress, the range, and forms of expressiveness of distress that are acceptable and adaptive in that context.

The cultural or contextual information includes the person’s understanding of the situation when it is applied to the diagnostic process of any mental disorders. This situation is either considered pathological by others in the person’s local world, or it is deemed to be normal. If it is considered pathological, further elements such as contextual information must be evaluated. These elements include the perceived level of severity of pathological symptoms, the dependence of the symptoms on the particular situation, and the relationship between the persistence of the distribution and the availability or non-availability of supports and interventions contributing to those symptoms. Thus, the cultural or contextual elements maximize the validity and usefulness of the diagnosis. Previously, mental disorders have persistently been reduced to the symptom lists without considering any contextual information. This exclusive reliance on clinical symptoms as the primary definition of mental disorders and its limitations stresses the importance of the inclusion of cultural and contextual factors in the Diagnostic and Statistical Manual of Mental Disorders- 5^th^ Edition (DSM-5, American Psychiatric Association, 2013). Currently, DSM-5 includes a Cultural Issues Subgroup that recommends the importance of concepts of culture in several areas of the manual. An introductory chapter on Cultural Aspects of Psychiatric Diagnosis was introduced comprising of a conceptual introduction, a revised outline for Cultural Formulation, a Cultural Formulation Interview, and a glossary on cultural concepts of distress along with the description of cultural issues in each disorder. This emphasizes the importance of understanding each disorder in the framework of culture.

### Culture and the Development of Personality (Normal or Abnormal)

Contextual information is crucial for understanding the development of personality. According to First et al. (1997), as cited from DSM-IV “Personality traits are enduring patterns of perceiving, relating to and thinking about the environment and oneself. They are exhibited in various important social and personal contexts. When these traits are significantly maladaptive and cause serious functional impairment or subjective distress, they constitute a personality disorder (PD)” (American Psychiatric Association, 2000, p. 686). PDs are often manifested in early adulthood. The diagnosis of a PD and the definition of a “normal personality” constitute a cultural and social construct. Culture defines the self, directs the orientation of the person toward the individual of the social group and defines how a normal personality is constructed and expresses itself in the social world. This implies that the difference between the normal and abnormal personality depends on cultural context, which sets the background in which an individual’s personality is developed. The concept and definition of PDs, as given in DSM, is based on the western notions of the individuals as “unique and independently functioning”. The application of this notion to people from cultures having different definitions of their normal functioning is thus questionable.

The relevance placed on behavior that is considered as deviant may be related to whether an action is seen as threatening to the prevailing social order (Cohen, 1972). Socially appropriate and acceptable behavior is variable over time and between cultures. Young (1990) has presented views on socially deviant and socially acceptable behaviors referred to as *cultural imperialism*. According to him, cultural imperialism is a social process by which values, experiences, and culture of the dominant group are viewed as a universal norm while groups or individuals who vary from this group are considered as inferior, deviant, or inadequate.

Formulations on the interplay of abnormal behavior and culture were also discussed by Tanaka-Matsumi and Draguns (1997). They highlighted the contextual analyses of psychopathology and the links between the major dimensions of culturally differentiating behavior (such as the dimension of individualism-collectivism and the self-orientation aspect of interdependent-independent self) and modes of expression of psychopathology. According to them, culture influences how individuals manifest symptoms, communicate their symptoms, cope with psychological challenges, and their willingness to seek treatment. Culture influences mental health in many ways (Castillo, 1997), which includes (a) how individual experiences and expresses his/her illness and the associated symptoms within the context of cultural norms, and (b) how the expressed symptoms are interpreted, diagnosed and treated. This view on culture and psychopathology is also emphasized by Gone and Kirmayer (2010), who asserted that contextual shaping of psychopathology is done by defining various sources of suffering which lie in a person’s local world or in his/her cultural identity. According to them, distinct forms of psychopathology depend to varying degrees on social context for their shape, content, and ‘natural history’. These forms include culturally mediated ways of understanding and interpreting an individual’s suffering. Researchers have identified the role of multiple collective influences that shape a person’s identity or his/her local world (e.g., Crenshaw, 1991; Iyer et al., 2008). The origin of such influences is conceptualized in an intersectional perspective and include nationality, migration status, racial and/or ethnic origin, language, religion, spirituality, moral traditions, age, gender identity, sexual orientation, socioeconomic and educational class, and functional status. Gold and Gold (2014) have also identified some more determinants of psychopathology that are engrained in the culture. These include urbanization, forced migration, climate change, war, political violence, new information technologies, and social media, giving rise to new forms of identity. Hassim and Wagner (2013) have specifically identified how culture and religion as a socio-contextual determinant of psychopathology define the acceptability of affect, cognition, and connation (see Lohani et al., 2013). Dein and Dickens (1997) have cited an example of the Muslim view on suicide, which is forbidden in Islam but is honored in some Japanese communities. Culture and socio-contextual factors, such as minority stress, discrimination, and exposure to interpersonal violence, influence the development of clinically significant distress and disability.

Cultural relativists and emic investigators were not in support of the applicability of western diagnostic criteria in other cultures (Hinton and Kleinman, 1993). There is evidence of cross-cultural differences in diagnostic practices, which influence diagnosis, the selection of treatment, and communication with the patients (Westermeyer, 1987). Mezzich et al. (1999) identified the impact of culture on psychiatric assessment and diagnosis, in at least five ways:-

(1) Culture shapes and directs the content, meaning, configuration, and the phenomenology of symptoms.
(2) Ethnopsychiatric diagnostic rationales as well as practices of grouping symptoms into patterns, including common culture-bound syndromes found in societies around the world.
(3) Culture provides a matrix for the interpersonal situation of the diagnostic interview.
(4) Since the clinical encounter is often inter-cultural, the dynamics of cross-cultural work are crucial for understanding and refining diagnostic categories and practices.
(5) Culture gives overall conceptualizations of the diagnostic systems, which usually become the products of their time and circumstances.

The DSM-IV (American Psychiatric Association, 1994) was the first acknowledgment of the impact of culture on psychiatric diagnosis (Christensen, 2001). The very definition of PDs in DSM-IV-TR (American Psychiatric Association, 2000; p. 686) emphasizes “PD is an enduring pattern of inner experience and behavior that deviates markedly from the expectations of the individual’s culture.” Despite the importance of culture, much of the theory and research about PD have severely underestimated or even ignored the influence of social organization and culture. According to Lewis-Fernandez and Kleinman (1994), personality and psychopathology take form in distinct local worlds where they are constructed by other individuals and the whole community and its institutions. Indigenous views and concepts of distress are considered fundamental to understanding the cultural context of illness (Angel and Thoits, 1987). According to Calliess et al. (2008), the extent to which PDs are influenced by cultural context is not known. Zimmerman (2015) has illustrated that borderline personality disorder (BPD) is considered as an under-researched disorder as compared to other psychiatric conditions. The review on culture influencing personality functioning and psychopathology emphasizes the importance of culture in the development of different psychological disorders. Since BPD is less researched about its development and manifestation, though it has an increasing prevalence, it becomes important to understand the disorder through the lens of culture.

To explore the influences of culture and psychopathology and then specific mental disorders such as BPD, a thorough literature search was done on the topics of culture, psychopathology, mental illness, cultural influences on personality development, development of abnormal personality, dysfunctional personality, personality disorders and culture, personality disorders in eastern and western cultures, cultural differences in PDs, Types of PDs influenced by culture, BPD and culture, BPD in India, and Indian context and BPD research. Many references were also searched from useful resources. These topics were searched alone and in combination with other topics. Around 800 research studies were found, while only 300 turned out to be useful in citing the researches and forming a general perspective or outlook of the current research.

## Culture and BPD: Culture and Social Norms Influencing BPD Features

According to Ronningstam et al. (2018), each culture has a specific history, values, and practices that may influence an individual, family, or group in such a way that affects personality functioning. Personality pathology is reflected in the breakdown of these cultural or societal norms. Hence, the diagnosis and treatment of PDs should consider the individual in the context of his/her culture. Cultural norms and context influence explicitly personality problems like emotion dysregulation and interpersonal hypersensitivity, which is the core characteristics of borderline personality disorder (BPD), the most common type of PD. BPD is one of the most common and widely studied of all the PDs described in the DSM-IV (American Psychiatric Association, 1994). Patients with this disorder have difficulty and impairment in their day to day functioning of life. They suffer from dysfunctional personality patterns causing severe distress to themselves as well as to those who are close to them. Some studies outlined that BPD is an excellent example of the many interactions between culture and Psychopathology (Alarcon and Foulks, 1995; Hughes and Wintrob, 1995).

In the case of BPD, the DSM-IV does not include the impact of misperceived acculturation, passivity, suicidal like behavior, identity, adjustment issues, and religious traditions on the presentation of people who resemble BPD (Paris, 1991; Alarcon, 1996). While in DSM- 5, the Cultural Formulation Interview (CFI) stresses on how the aspects of individuals’ background, developmental experiences, and social contexts can affect their perspective on the psychiatric condition, e.g., the cultural definition of a problem leads the patient to seek treatment or not to seek it. Cultural perception of the cause of a problem, context, and available support can influence treatment motivation also (DSM-5, pp. 750–754). Many researchers have emphasized that religion, spirituality, or moral standards are aspects of culture that may influence psychopathology (e.g., Crenshaw, 1991; Dein and Dickens, 1997; Iyer et al., 2008; Hassim and Wagner, 2013). Although in the case of BPD, it plays a less significant role. As demonstrated by Hafizi et al. (2014), religiosity and religious attendance are negatively correlated with borderline traits (like anger, unstable mood, feelings of emptiness, and self-harming behaviors). Hence it is important to explore various socio-contextual determinants that may influence the development of BPD. Indian cultural context has been used to illustrate the importance of these socio-contextual determinants.

### Social Norms and Interpersonal Functioning

In Axis II of the DSM, PDs are defined based on how an individual considers whether someone’s personality deviates from the general social norms and characteristics. Foulks (1996) emphasized that the “culturally relative exercise” of labeling usually deviates from ‘normal personality’ about specific values, ideas, world views, resources, community size, and social structures. An example of this culturally relative exercise of labeling is seen in Asian and western cultures. Asians place a higher value on the interdependence between interpersonal relationships, whereas western cultures emphasize separation and independence. An illustration of normal vs. psychopathological interpersonal functioning can be seen in Indian family settings, particularly in extended or larger families where frequent arguments and fights among its members are viewed as normal, whereas such expressions may be considered as a symptom of BPD by a western therapist. Family members continue to live with each other despite stormy relationships, so the abandonment fear is not a common feature in eastern cultures like India. In an interpersonal context, BPD patients try to control or gain support from significant others by showing manipulation, intimidation, and bullying. According to Gunderson (1984), the manipulation includes somatic complaints, provocative actions, misleading messages, and self-destructive acts. Therefore, the context has the potential to trigger such manipulative acts. These acts are perceived as normal unless it harms the individual or significant others. Indian family structure is tolerant of such manipulative acts up to a greater extent as compared to the western countries. Thus, the context nurtures, or harbors certain manipulative acts and do not consider it as deviant unless it disrupts other significant areas of functioning other than interpersonal functioning. Hence, relational manipulation is a common feature instead of abandonment fears and stormy relationships, which is seen as pathological if it disrupts significant areas of functioning. Researchers have found that family is an important resource for the care of mental health problems, but its role in the management of any mental health condition is minimal (Chadda and Deb, 2013). They have also emphasized that family may be a source of trouble or support to the individual. According to Johnson (1995), families serve as agents to transfer the cultural values in its members. These views on Indian families highlight the fact that a family provides a context for the development of BPD features, nurtures and promotes the deviant manipulative and impulsive acts, and protects certain other features like abandonment fears and stormy relationships. Thus, it is important to look at the family values and functioning while assessing the interpersonal features of BPD.

Interpersonal functioning is also influenced by the type of relationship one has with his/her family members. Sinha and Sharan (2007) have illustrated that early attachment failure or insecure attachment with the authority figures leads to the development of another BPD feature such as intolerance of aloneness and dependency on others. Meyer et al., 2001 further explains that individuals with BPD usually have ‘ambivalent and erratic feelings in close relationships’ as they fear the loss of attachment figure as well as they long for dependency. Here loss of attachment figure does not give rise to abandonment fears; instead, it emphasizes dependency. This leads to a splitting reaction of BPD patients in their interpersonal relationships.

### Emotions in Cultural Context

Individuals with BPD often co-occur with other mood/affective disorders (Klein, 1977; Gunderson, 1984; Stone, 1986; Johnson, 1991; Judd and McGlashan, 2003). Expression of strong feelings is culturally discouraged, so they are usually expressed psychosomatically in terms of headaches, stomach problems, etc. (Liu et al., 2004). Feelings of hurt, blame, self-loath, hopelessness, helplessness, and being unlovable are associated with BPD features whenever an interpersonal crisis occurs. Therefore, these strong feelings lead to a strong need for emotional support and nurturance to fulfill one’s affectional needs. The dynamic social and family systems provide emotional support and nurturance. If this system lacks support, these feelings are unmet and result in an emotional crisis or breakdown, thereby leading to the experience of BPD features. On the other hand, this system may inhibit or facilitate the overt expression of such strong feelings indicative of BPD. Hence, the protective social system and cultural factors help in the experience and overt expression of emotions, which may be strong enough to give a diagnosis of BPD.

### Self and Culture

Psychological notions such as self-concept and self-image must be taken into consideration as they are strongly influenced by cultural forces. The mental representation of self is influenced by the surroundings in which the individual grows up and interacts with. According to Markus and Kitayama (1991), individuals carry a different sense of self concerning how it is related to significant others across cultures. Lalonde et al. (2004) have emphasized that Asians tend to develop an interdependent self, which is a more fluid and flexible view bound to others. Cheung (1998) has stated that selfhood in eastern cultures includes interpersonal constructs, thereby influencing identity, which cannot be separated from significant others. Development of one’s identity takes place in a social context; thus, culture and social structures affect identity formation and its diffusion. Again, the role of the family in transcending the values of sense of self and the identity is minimal, or identity of an individual is interdependent on significant others and is usually studied in the context of interpersonal relationships.

## Cultural Differences in BPD

BPD occurs in many different cultures around the world (Loranger et al., 1994). Many studies present researches on BPD in their own cultures. Since there is much debate in eastern and western cultures, the focus of the present study is more on citing evidence from eastern cultures. Currently, there is a low diagnostic prevalence of BPD in Asian cultures, and few studies on BPD phenomena using Asian populations, particularly in India, exist. Examples of cultural variations can be seen in studies of Beiser (1987), who emphasized that extremes of idealization and devaluation can be fostered by cultures where authority figures are revered without any questioning. The diagnosis of BPD in the Chinese psychiatric community contended that some of BPD’s criteria, such as fear of abandonment, are not appropriate in the Chinese cultural context, which values collectivistic identities and enmeshed relationships (Yang, 1986; Chinese Society of Psychiatry, 2001). In India, a meta-analysis of 13 psychiatric epidemiological studies (n = 33, 572) yielded an estimated prevalence rate of organic psychosis, alcohol/drug dependency, schizophrenia, affective disorders, neurotic disorders, mental retardation, and epilepsy without reporting any prevalence of PDs (Reddy and Chandrasekhar, 1998). PDs in India appear to be less researched and under-diagnosed disorder. This suggests how the influence of culture can affect the development of symptoms (Hwang et al., 2008), which are less prevalent or less common in certain cultures and influence the course of many mental health problems (Marsella and Yamada, 2010), including PDs (Ziegenbein et al., 2008). Thus, the present paper further extends its focus on reviewing the researches in the Indian cultural context specifically on BPD, its manifestation, and thereby drawing conclusions based on the review concerning this particular culture. Although cultural influences are seen in almost all mental health conditions, the development of a personality that is psychopathological is, in itself, a cultural phenomenon. Hence, how BPD develops in India, where the family is an important medium of transcending cultural values, needs to be studied.

## Researches on BPD in India

Research on PDs is a growing field in India as it was less researched in the 1990s, but the number of studies kept increasing. Many research focused on studying PDs in general; however, very few of these targets the specific subtypes of PDs such as BPD. Moreover, studies on BPD in the Indian context are limited. A summary of BPD research in India has been presented. These researches have used inconsistent measures to identify BPD cases such as Case histories, DSM Criteria, symptoms checklist, or translated versions of scales which have been used in the western countries. Some studies have found the occurrence of BPD while others have reported minimal research indicating the need for more inquiry into this growing field. For example, a survey by Latha et al. (1996) has presented a prevalence rate of PD, which was 12% without any incidence of BPD. Earlier studies focusing on the occurrence of BPD in India have rarely reported significant information about the prevalence of the disorder. In an early study, Paris (1996) used a case of a patient of Indian descent who developed BPD after she immigrated to Canada. The author elaborated on the hypothesis that BPD appeared to be highly sensitive to the socio-cultural context and asserted that risk factors underlying BPD exist in developing countries. He further stated that some traditional cultures, like India, provide protective factors that suppress the overt expression of BPD symptoms. Other researches have mainly focused on the study of suicide and its comorbidity with PDs, including some cases reporting the prevalence of BPD. One such study by Gupta and Trzepacz (1997) has emphasized the characteristics of suicide attempters who overdosed poison and were admitted to a general hospital. These individuals were compared with those who used other methods such as non-overdose and with medically ill patients having suicide ideations only. They reported that all the groups had sought psychiatric services earlier. Each group met the DSM-III-R (American Psychiatric Association, 1987) diagnoses of depression, adjustment disorders, and substance abuse, while the overdose group exhibited significantly more borderline features and had more female patients.

Some studies on BPD in India have begun to focus on its relationship with other psychological constructs and its comparison or comorbidity with these disorders. There were still very few reports of BPD from India. This led to a study of a small sample from a very narrow segment of suicide attempters who presented to a charitable hospital in a city. Out of 75 suicide attempters, 13 (8 Males and 5 Females) patients were later found to have BPD. This report on BPD in India confirms its high prevalence rate and that it may be under-diagnosed in the clinical setting (Pinto et al., 2000). However, in another study, the application of the International Personality Disorder Examination (IPDE) led to the emergence of emotionally unstable PD (impulsive and borderline type) as the most common PD (Sharan, 2001). Chandrasekaran et al. (2003) found that among 341 patients who attempted suicide, PD was identified only in 7%, with 0.58% cases of BPD and all suffered from a comorbid psychiatric illness. Nath et al. (2008) aimed to identify the type of PD commonly associated with deliberate self-harm. It was found that the most common disorder was emotionally unstable (both borderline and impulsive type, 28.6%) in young females.

In a review of 13 studies on attachment and BPD, a strong association between insecure forms of attachment and BPD was observed (Agarwal et al., 2004). These patients informed about the lack of availability of attachment figures; they were afraid to lose attachment figures; they lacked the use of these figures, and protested separation (Sinha and Sharan, 2007). Aaronson et al. (2006) have reported that patients with BPD were more likely to exhibit (vacillate between) angry withdrawal and compulsive care seeking (which is a form of securing attachment). A study by Belhekar and Padhye (2009) explored the relationship of BPD with affective instability and Five-Factor Model’s neuroticism and found affective instability as a core component of BPD.

Newer studies by Gupta and Mattoo (2012) have identified the prevalence of PDs among psychiatric outpatients and reported that such incidence was very low as compared to the research literature. The sample had a PD prevalence of 1.07% and contained more students or unemployed single young men. Borderline and anxious-avoidant PDs were the most common. The borderline group was younger (mean age 24.44 years), had more women (60%), housewives (28%), and had patients more with a lower-income background (80%). Another study by Mitra and Mukherjee (2013) explored whether bipolar affective disorder and BPD fall under the same spectrum, or they represent separate categories. Features of immaturity and instability were common in both bipolar affective disorder and BPD. The Probable pathways of the development of psychopathology in these two disorders were explored.

Sarkar et al. (2016) have found a link between PDs and other psychiatric co-morbidities common in the setting of severe acne. In many patients, emotional stress and psychological problems such as social phobias, low self-esteem, or depression may occur as a result of acne. In a total of 65 patients, PD was present in 29.2% of patients. The diagnosed PDs were obsessive-compulsive personality disorder (*n* = 9, 13.8%), anxious (avoidant) personality disorder (*n* = 6, 9.2%), and BPD (*n* = 2, 3%), mixed PD (*n* = 2, 3%). All patients with PDs had some significant psychiatric comorbidity like anxiety and depressive disorders.

Case studies on BPD have also been done to gain a deeper understanding of the presentation of the symptomatology. Duggal and Fisher (2002) presented a case of repetitive tattooing, a kind of self-mutilating behavior described in a patient with BPD with a co-morbid obsessive-compulsive disorder (OCD). Tattooing was viewed as a self-mutilating act, which is characteristic of BPD. Notably, tattooing initially represented an act of self-mutilation in consonance with the underlying PD. Self-mutilating behavior has also been discussed in a case report presented by Mago (2011). The case expressed an aggressive component directed against himself, which can specifically be explained by his low self-esteem and insecure personality structure, particularly BPD. Another case study presented a BPD patient with difficulties in living and poor living conditions and quality of life. An intervention for enhancing his quality of life was proposed (Choudhary and Thapa, 2012). Some more cases reported the clinical profile of BPD patients with specific features such as suicidal tendencies, substance abuse, disturbed emotions, and difficulty in controlling emotions. A dialectical Behavior Therapy Intervention plan was suggested to the patient showing such features who eventually was a dropout (Choudhary and Thapa, 2013). A study by Choudhary and Thapa (2014a, b) has examined specific features in BPD patients in 8 cases of BPD. A list of characteristics defining BPD was found viz, substance abuse, suicidality, academic failure, social dysfunction, dependency on others, and subjective personal distress. The findings also indicated that significant areas of patients’ life were impaired. The clinical population in India encountered a major problem as reported in this study was the family attitude, which served as a major barrier in seeking therapy or professional help. Narayanan and Rao (2018) also illustrated that the family acted as both a source of stress and abuse as well as support. Another case, reported as a letter to the editor, described a female patient having WattsApp (a popular smartphone messenger application) addiction and BPD. BPD characteristics such as the feeling of emptiness, getting bored easily, and unstable self-image increases the proneness for WhatsApp addiction as these people may use mobile more often to stay in touch with a greater number of persons (Faye et al., 2016). There is also evidence of dependent Internet users ranking high in the feeling of loneliness, affective disorders, low self-esteem, and impulsive behaviors, which are common in BPD (Beranuy et al., 2009). This case signifies that WhatsApp addiction emerges as an important behavioral addiction having negative consequences, and BPD is a risk factor in developing WhatsApp addiction. Choudhary and Thapa (2017) found a characteristic profile for evaluating the emotional and cognitive functioning of BPD using a case study and mental status examination of 5 cases of BPD. Direct observation and in-depth clinical interviews of patients rendered characteristic themes such as:-

1. General appearance, which was appropriate, indicating males as dull, sad and reclusive and females as elated. 2. Psychomotor activity and speech of patients were slow, increased in some cases. 3. The emotional state of these patients included feelings of anger, hurt, boredom, and depression. 4. The affective state was not compatible with the idea and content of thoughts of these patients. 5. Suicidal ideations were common in all patients. 6. Sensorium and mental capacity of patients indicated that their remote memory was adequate, recent memory was impaired, and immediate memory was poor. 7. Attention and concentration of patients were poor, and general awareness was average. 8. The insight and judgment capacity of patients revealed that they were aware of the existing mental problem and urged for their problems to be shared, heard, and understood by others.

A review paper on Indian scales and inventories identified several measures for assessing different aspects of human behavior and personality (Venkatesan, 2010). This paper highlighted the Indian adaptations of several measures evaluating personality, cognition, social aspects, and diagnosis of psychiatric illness with very few measures for identifying specific mental disorders Such an attempt of adaptation of measures for identifying BPD was done on a very small sample of BPD patients by Choudhary (2017). In the study, three scales (MCMI-III Grossman Facet scales (Millon et al., 1994, 1997, Grossman and Del Rio, 2005), PAI-BOR (Morey, 1991) and MSI-BPD (Zanarini et al., 2003) were selected for cultural adaptation and validation. These three scales were found to be efficient in diagnosing BPD and can be reliably used to diagnose BPD in India, although its generalizability is questionable as the sample size was small for validation.

## Implications for Research on BPD

BPD is a prevalent and highly controversial mental disorder that lacks consensus on assessment and diagnosis. Diagnosis, if not done correctly, can lead to ineffective treatment, thereby causing potential harm to the patients. The increasing rate of diagnosis of BPD in the Indian context focuses on more emerging researches on BPD. BPD was initially found to occur in approximately 1-4% of the general population (Crawford et al., 2005; Coid et al., 2006; Lenzenweger et al., 2007), and about 75% were women (Widiger and Weissman, 1991; Skodol et al., 2002). But in a recent study by Grant et al. (2008), the lifetime prevalence of BPD in the general population rose up to 6%, although in different countries, it is present with different prevalence rates, so the exact rate is debatable. Hence, the general population between 1.2% and 6% is estimated to meet the diagnostic criteria for BPD, and this cohort comprises approximately 10-15% of individuals using outpatient psychiatric services and approximately 20% of those in inpatient psychiatric care (Levy et al., 2006; Crowell et al., 2009).

The review of Indian researches asserts that previous studies have reported the existence of BPD in India, with protective factors that suppress the overt expression of its symptoms. After that, the prevalence of a PD (specifically BPD) was mainly identified in suicide attempters. High co-morbidity of people with deliberate self-harm and BPD was found. Prevalence of BPD was studied in newer researches where some socio-cultural factors such as unemployment, younger age, low socio-economic status, and being women were found to be associated with BPD. Some other studies focused on the co-morbidity of BPD with bipolar disorders. A link between severe acne and BPD was also found. Case studies have been done to gain a deeper understanding of the presentation of the disorder. Repetitive tattooing was shown with the presentation of OCD and BPD. Cases of BPD were presented with the clinical profile of BPD having specific features such as suicidal tendencies, substance abuse, disturbed emotions and difficulty in controlling emotions, problems in living, poor living conditions and poorer quality of life. Certain BPD characteristics were observed in case studies of patients, which included substance abuse, suicidality, academic failure, social dysfunction, dependency on others, and subjective distress. Impairments in areas such as intimate relationships and occupational functioning were also identified along with a major problem in family functioning and their attitudes which abstained them from seeking professional help as well as in the overt expression of symptoms. Another case found a link between BPD symptoms such as emptiness, boredom, and unstable self-image and WhatsApp addiction. Recent case study delineated the emotional and cognitive functioning of BPD patients. Extreme emotions with mood swings and mild cognitive distortions were found in BPD. However, the synthesis of Indian studies presents the defining characteristics, prevalence, and comorbidity with suicide attempters, Bipolar Disorder, and OCD along with socio-cultural risk factors. Patients with BPD frequently have a complex presentation and are regularly co-morbidly diagnosed as compared to other diagnostic groups (Levy et al., 2006; Harned et al., 2008). This prevalence of co-morbidity complicates the diagnostic presentation of patients and, consequently, their treatment. This information on context can also be used for treatment planning. Therefore, these inferences call for a need to develop more specific tools keeping in mind the information gathered from the synthesis of Indian studies for the assessment and differential diagnosis of BPD.

Linehan (1993) reported that approximately 75–80% of borderline patients attempt or threaten suicide, and between 8–10% are successful. If the borderline patient suffers from depressive disorder, the risk of suicide is much higher. Therefore, early identification and appropriate interventions are crucial. The borderline diagnosis establishes a basis for developing a treatment alliance by offering patients a developmental and therapeutic context that they will experience as meaningful and appropriate. This information on context can also be used for treatment planning.

Since the characteristic features and presentation of BPD symptoms were analyzed only through case studies and a small sample of BPD patients, diagnostic generalizations are difficult to formulate in the context of culture. Formulating an accurate diagnosis of BPD in itself is difficult and is reported to have low reliability and validity, often resulting in misdiagnosis (Fiedler et al., 2004). The efforts to develop diagnostic measures were also not sufficient because the measures to identify BPD were only adapted, not developed, and were validated on a tiny sample of BPD patients. Hence, comprehensive studies in this area are needed. In-depth interviews and case analysis of BPD patients on large samples are required to attain diagnostic reliability, which will render a clinical picture of the disorder and will also be helpful in understanding the severity and complexity of the disorder.

Studies have also illustrated that structured interviews and questionnaires do not correlate strongly with consensus diagnoses made using available data collected over time by clinicians because the clinicians have direct access to data from other informants, and they know the patients very well (Pilkonis et al., 1991, 1995; Skodol et al., 1991). There is no single method that best assesses BPD. However, literature revealed that the Millon Clinical Multi-Axial Inventory- III (MCMI-III) and the Minnesota Multiphasic Personality Inventory-2 (MMPI-2) were the most widely used instruments to assess PDs in general, even though each tool is not without limitations and controversy on their validity from other researchers. Therefore, a multi-method approach to diagnosis using various diagnostic measures is preferred over the single method.

Various researches have illustrated that BPD occurs in many cultures (e.g., Loranger et al., 1994) and that the DSM diagnoses should not be utilized universally without considering the cultural aspects of the individual because the cultural context plays a significant role in shaping the development of a person’s personality. The cultural context often determines the presentation and manifestations of symptomatology. Culture provides a framework for our thoughts, beliefs, and behavioral styles that are specific to a particular society. BPD has a variety of features that may be specific to the Indian cultural context, so there is a need to study the culture-specific manifestation of the disorder. For example, self and identity are core features of BPD which have not been studied in the Indian context. There is a lot of debate in the development of self and identity in the Indian context, but the development of pathological self and identity as expressed in BPD has not yet been explored. Hence, the disorder should be examined comprehensively and in detail. In-depth interviews and case analysis of BPD patients will provide a clinical profile of the disorder and will help in the understanding of the complex clinical features that constitute the disorder within this cultural framework.

### Conclusion

This article is an analysis of how culture shapes normal and abnormal personality within the framework of a context or culture. A specific context influences patterns of thinking, feeling and behaving in ones’ local world. The paper further summarizes significant attempts of researches done on the culture and development of psychopathology in the east which further focuses on India. How BPD features like interpersonal functioning, emotion regulation and self and identity can be understood across cultures specifically in Indian context is also highlighted. The symptomatology or the presentation of symptoms of mental disorders has not been done comprehensively, so the diagnostic reliance on culture-specific symptoms cannot be formulated. Hence, the importance of culture in the description of mental disorders was emphasized as the symptoms develop, are manifested and maintained in the person’s context. A summary of major attempts to study the most common type of personality psychopathology that is borderline personality disorder was presented. The account of such studies reflects on the need to study the disorder in a comprehensive and detailed manner using cultural information. A comprehensive analysis of Indian studies indicates the directions for more research on BPD. Diagnostic considerations of BPD are needed in India as the disorder is gaining popularity.

## Author Contributions

All authors listed have made a substantial, direct and intellectual contribution to the work, and approved it for publication.

## Conflict of Interest

The authors declare that the research was conducted in the absence of any commercial or financial relationships that could be construed as a potential conflict of interest.
